# Who is where at risk for Chronic Obstructive Pulmonary Disease? A spatial epidemiological analysis of health insurance claims for COPD in Northeastern Germany

**DOI:** 10.1371/journal.pone.0190865

**Published:** 2018-02-07

**Authors:** Boris Kauhl, Werner Maier, Jürgen Schweikart, Andrea Keste, Marita Moskwyn

**Affiliations:** 1 AOK Nordost–Die Gesundheitskasse, Department of Medical Care, Berlin, Germany; 2 Beuth University of Applied Sciences, Department III, Civil Engineering and Geoinformatics, Berlin, Germany; 3 Helmholtz Zentrum München, German Research Center for Environmental Health (GmbH), Institute of Health Economics and Health Care Management, Neuherberg, Germany; Forschungszentrum Borstel Leibniz-Zentrum fur Medizin und Biowissenschaften, GERMANY

## Abstract

**Background:**

Chronic obstructive pulmonary disease (COPD) has a high prevalence rate in Germany and a further increase is expected within the next years. Although risk factors on an individual level are widely understood, only little is known about the spatial heterogeneity and population-based risk factors of COPD. Background knowledge about broader, population-based processes could help to plan the future provision of healthcare and prevention strategies more aligned to the expected demand. The aim of this study is to analyze how the prevalence of COPD varies across northeastern Germany on the smallest spatial-scale possible and to identify the location-specific population-based risk factors using health insurance claims of the AOK Nordost.

**Methods:**

To visualize the spatial distribution of COPD prevalence at the level of municipalities and urban districts, we used the conditional autoregressive Besag–York–Mollié (BYM) model. Geographically weighted regression modelling (GWR) was applied to analyze the location-specific ecological risk factors for COPD.

**Results:**

The sex- and age-adjusted prevalence of COPD was 6.5% in 2012 and varied widely across northeastern Germany. Population-based risk factors consist of the proportions of insurants aged 65 and older, insurants with migration background, household size and area deprivation. The results of the GWR model revealed that the population at risk for COPD varies considerably across northeastern Germany.

**Conclusion:**

Area deprivation has a direct and an indirect influence on the prevalence of COPD. Persons ageing in socially disadvantaged areas have a higher chance of developing COPD, even when they are not necessarily directly affected by deprivation on an individual level. This underlines the importance of considering the impact of area deprivation on health for planning of healthcare. Additionally, our results reveal that in some parts of the study area, insurants with migration background and persons living in multi-persons households are at elevated risk of COPD.

## Introduction

Chronic obstructive pulmonary disease (COPD) is a potentially preventable chronic respiratory disease, which is characterized by airflow limitation and is not fully reversible [1]. COPD has grown to be the 4^th^ leading cause of death worldwide [2] and is projected to be the third leading cause of death by 2020 [3]. The prevalence of COPD increases not only in low-income countries, but also in high-income countries with a growing proportion of elderly persons [3–5]. Due to the demographic transition in Germany, the prevalence of COPD is expected to grow by 24% in 2030 [5]. Cigarette smoking is the main risk factor for COPD [6], followed by in- and outdoor air pollution [7, 8], occupational hazards and respiratory infections [4].

Current prevalence estimates for Germany range from 1.3% to 13.2%, depending on the population included, definitions for COPD used and method of data collection [5, 9]. The economic burden of COPD on the German healthcare system is high as treatment for COPD is very cost-intensive and is associated with a high chance of work impairment [10, 11]. However, the majority of studies estimating the incidence or prevalence of COPD in Germany rely on voluntary participation of individuals through surveys or questionnaires [9, 12, 13]. Although these studies provide important insights about risk factors on an individual level, the results have only very limited use for a demand-based planning and allocation of healthcare and targeted prevention strategies.

Geographic information systems (GIS) facilitate the analysis of disease heterogeneity and allow the identification of high-risk areas. This is important to allocate financial resources for healthcare and targeted prevention strategies where they are needed most [14, 15]. Analyzing risk factors on an aggregated population-level rather than individual level does not only leads to results, which are more generalizable for the whole population [14], but also helps to model the future expected demand for healthcare [16]. This background knowledge is especially valuable for health insurance providers in the context of the German healthcare system.

As planning and allocation of primary healthcare in Germany is organized between the association of statutory health insurance physicians and the respective health insurance providers, detecting areas with increased demand for healthcare and understanding population-based processes associated with this increased demand is important to provide healthcare where it will be needed most. Health insurance enrollment is mandatory in Germany with 86% of the population being covered by a statutory health insurance provider, 10% being covered by a private health insurance provider and the remaining 4% being covered by the state [17, 18]. As a result, statutory health insurance databases can provide a comprehensive overview about the prevalence of COPD and other chronic diseases in a large sample of the population. However, it is important to note that large differences in the demographic and socio-economic composition of the members of various statutory health insurance providers exist, with the Allgemeine Ortstkrankenkasse (AOK) having the largest proportion of persons with a lower socio-economic status and thus the highest prevalence of chronic diseases [18–21]. Logically, population-based risk factors for chronic diseases may vary to an extent among different health insurance providers and it is important for each health insurance provider to analyze possible risk factors in relation to the demographic and socio-economic composition of their members to be able to engage in evidence-based negotiations, where new GPs should be allocated.

Although previous GIS-based studies have clearly shown that COPD is highly heterogeneously distributed across space [15, 22, 23], similar studies are currently unavailable in Germany and the spatial distribution and population-based risk factors of COPD therefore still remain unknown. Previous studies based on data of northeastern Germany`s largest health insurance provider have clearly shown that chronic diseases such as type 2 Diabetes Mellitus and hypertension are not only highly heterogeneously distributed across northeastern Germany, but also that the association to possible risk factors underlies strong regional variation [18, 20, 24]. As COPD is a frequently diagnosed disease among members of the AOK Nordost, a spatial epidemiological approach will help to inform evidence-based planning and allocation of healthcare, targeting those population groups, which are most at risk in specific locations.

The aim of this study is to (i) examine the spatial distribution of COPD prevalence at the smallest spatial scale possible based on health insurance claims of the AOK Nordost, (ii) identify population-based risk factors and (iii) analyze how these associations varies across northeastern Germany.

## Methods

### Dependent variable

The AOK is with over 24 million insurants Germany`s largest statutory health insurance provider and covers 34.9% of all 69 million statutory health insurants in Germany [25]. In contradiction to other statutory health insurance providers in Germany however, the AOK is divided into 11 local AOKs. The data source for this study stems from the AOK Nordost, which is the 6^th^ largest AOK with regards to the number of persons insured [26]. The AOK Nordost is the largest statutory health insurance provider in northeastern Germany and covers roughly a quarter of the population in the federal states of Berlin, Brandenburg and Mecklenburg-West Pomerania. A description of the demographic characteristics of the AOK Nordost insurants is provided in Table 1. Of the 1.79 million insurants, 149 thousand (8.3%) were diagnosed with COPD. We defined COPD as a confirmed diagnosis with the ICD-codes (10^th^ revision) J44.

**Table 1:** Demographic composition of the AOK Nordost insurants in 2012.

Age	Female	Male
0–4	1.77%	1.89%
5–9	1.68%	1.77%
10–19	3.31%	3.50%
20–24	2.48%	2.54%
25–44	8.92%	9.88%
45–64	12.22%	14.37%
65–79	12.29%	10.17%
> 80	9.47%	3.78%

As long as an insurant is treated for COPD, this diagnosis remains in the insurants personal medical file. The unique insurant number was used to ensure that each insurant is included only once in the analysis [18, 24].

We aggregated the COPD health insurance claims to Northeastern Germany`s municipalities and within cities to the urban districts and postal codes to visualize the sex- and age-adjusted prevalence. As the municipalities between Brandenburg and Mecklenburg-West-Pomerania vary strongly in size and inhabitants, we considered the municipality-level as not suitable for the spatial regression analysis. We therefore aggregated the health insurance claims to the association of municipalities (Gemeindeverbände), which is the next-smallest spatial scale available [24]. The underlying map sources for the municipalities and the association of municipalities were downloaded from the federal agency of cartography and geodesy [27].

### Explanatory variables

For the regression analysis of COPD prevalence, we used a wide range of possible explanatory variables. Based on the insurance database, we used the proportion of insurants aged 45–64 years,65 years and older as well as the proportion of insurants with migration background. To measure the influence of a lower socio-economic status on COPD, we used the German Index of Multiple Deprivation (GIMD), which was obtained from the Institute of Health Economics and Health Care Management at the Helmholtz Zentrum München, German Research Center for Environmental Health. The index consists of seven different domains of deprivation (income, employment, education, municipal revenue, social capital, environment and security) [24, 28, 29]. The original index was available for the municipalities in Germany and was aggregated to the association of municipalities to match the dependent variable of the regression analysis.

Further explanatory variables related to marital status, air pollution, availability of healthcare and household composition were taken from the Census 2011 of Germany (Table 2).

**Table 2:** Possible explanatory variables.

Variable	Year	Data source
AOK Nordost insurants aged 45–64 (%)	2012	AOK Nordost database
AOK Nordost insurants aged 65 and older (%)	2012	AOK Nordost database
Insurants with migration background (%)	2012	AOK Nordost database
Area deprivation	2010	Helmholtz Zentrum München
Married persons (%)	2011	Census 2011 for Germany
Unmarried persons (%)	2011	Census 2011 for Germany
Divorced persons (%)	2011	Census 2011 for Germany
Widowed persons (%)	2011	Census 2011 for Germany
Average household size	2011	Census 2011 for Germany
One-person-households (%)	2011	Census 2011 for Germany
Average distance to GPs	2012	AOK Nordost database
Inhabitants per GPs	2012	AOK Nordost database
Fine particulate matter	2012	Environment research institute
Traffic load	2012	federal highway research institute
Average distance to highways and main streets	2012	OpenStreetMap

### Statistical analysis

#### Cartographic visualization of sex- and age-standardized prevalence rates

As our goal was to visualize the spatial distribution of the sex- and age-adjusted COPD prevalence, we used the German population of 2011 in different sex- and age-groups as standard population, which was obtained from the census 2011 for Germany [30]. Since not only the number of inhabitants varies greatly within the munipalities and urban districts, but also the proportion of members of the AOK health insurance, we applied the conditional autoregressive Besag-York-Mollié (BYM) model without covariates to account for the strongly varying population densities to generate more stable and reliable prevalence rates. In it`s basic form, the BYM model is a Poisson model where the number of sex- and age-adjusted number of COPD cases is the dependent variable and the total number of AOK Nordost insurants is the offset variable. The BYM model then weights the prevalence rate of a specific municipality towards the prevalence of neighboring municipalities [31]. The neighborhood structure was defined as areas sharing a common edge or border [31, 32]. We first fitted the model with minimally informative priors specified on the unstructured and structured effects with a precision of logGamma (1, 0.0005), but run the model also with different precision parameters to evaluate in how far the choice of prior distribution has an effect on the posterior distribution of the prevalence estimates [33]. Bayesian disease mapping models are often based on Markov Chain Monte Carlo (MCMC) simulations. However, MCMC calculations are often very time-consuming and convergence of the simulations is often unpredictable. The integrated nested laplace approximation (INLA) was developed to overcome the limitations associated with MCMC simulation. The calculation of the BYM model was therefore carried out using the R package “INLA” [34].The results were then imported in ESRI ArcGIS 10.2.

#### Local cluster analysis

To pinpoint areas for future public health interventions, we used the spatial scan statistic (SaTScan) [18, 35]. The spatial scan statistic identifies the location and statistical significance of possible clusters [36]. We used a purely spatial Poisson model, where the number of COPD cases is expected to follow an inhomogeneous Poisson distribution [36]. The number of sex- and age-adjusted cases, the number of total insurants and the centroid coordinate of each administrative unit was used as input for this model. The spatial scan statistic uses a circular scanning window, which is flexible in size and position and moves over the coordinates of the study region and in our study evaluates all possible cluster positions and sizes up to a used defined radius of 10km at most. This setting helped to detect spatial clusters as precise as possible. 10km were defined as the maximum bearable driving distance to a GP [18, 24]. The statistical significance was evaluated using 9,999 Monte-Carlo replications. We considered only clusters with a p-value <0.001 [18, 24]. This was done as a p-value of 0.05 could theoretically still detect 72 of the 1,449 administrative areas as statistically significant clusters although they constitute only false positives. A very conservative p-value of 0.001 in contradiction, would detect only one area as statistically significant false positive.

#### Geographically weighted regression modelling

Based on previous research in the study area, we suspected that a global model for COPD–similar to previous studies on type 2 Diabetes Mellitus [18] and hypertension in this area [24]–will have a relatively modest explanatory power. We therefore chose to identify important explanatory variables directly in a geographically weighted regression (GWR) model. For this task, we used the R package “GWmodel” [37]. To satisfy the assumption of a normal distribution of the dependent variable for a Gaussian GWR, the COPD prevalence was log-transformed. In the next step, we used GWmodel`s “model.selection.gwr” function. This function is comparable to a forward step-wise regression: In each step, one of the not yet included explanatory variables is added to the GWR model and the resulting AICc values are compared so that the variable resulting in the lowest AICc value remains included in the selection process. This step is repeated until all explanatory variables are included in the model. The resulting models were then ordered by their AICc values. We then chose the final model with the lowest AICc value and the most plausible explanation of COPD prevalence and tested for local multicollinearity using the “gwr.collin.diagno” function in the GWmodel package. Multicollinearity can be measured by the variance inflation factor (VIF). A value >10 indicates local multicollinearity [38]. However, the choice of kernel function and bandwidth size has a considerable effect on the performance of GWR [39]. We therefore repeated the identification-process of explanatory variables with several combinations of kernel function and bandwidth specification. To ultimately find the best fitting model, we evaluated all possible kernel functions, bandwidth options and optimization methods in GWmodel. The form of the kernel can be specified as either Gaussian, bisquare, tricube, exponential or boxcar. The bandwidth can be either defined by specifiying a number of observations to be included in the kernel or a fixed bandwidth with a fixed radius in km. These parameters can be optimized by either Akaike`s corrected information criterion (AICc) or cross validation (CV). All possible combination possibilities can then be compared against each other by their AICc and adjusted R^2^ values, to find the best fitting model. Additionally, GWR calculates a global model to test the hypothesis that a local model provides a better fit than a global model. For the basic GWR, 20 different combinations of kernel form, bandwidth and optimization method were evaluated [37]. Clustering of the residuals was evaluated using the spdep package in R version 3.3.1 [32]. The results were then imported for visualization in ESRI ArcGIS 10.2.

### Ethics statement

The data and results used in this study were anonymized and do not contain any personal information. The use of anonymized data for research purposes does not require a vote by an ethics committee or an institutional research board.

## Results

### Spatial distribution of COPD

**Fig 1:**
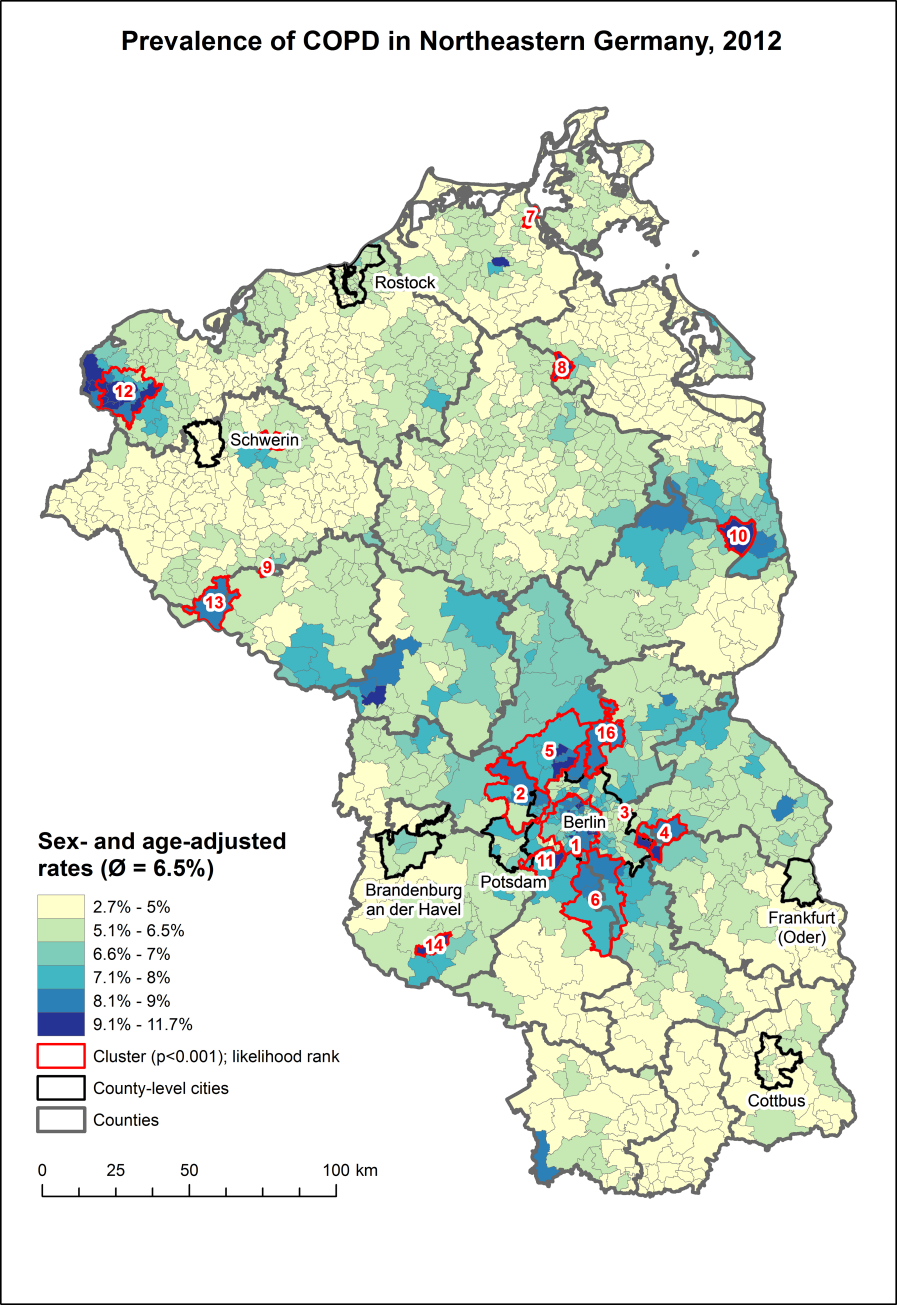
Posterior mean of the sex- and age-adjusted prevalence of COPD across municipalities and urban districts in northeastern Germany.

The overall sex- and age-adjusted mean posterior prevalence of COPD was 6.5% among the AOK Nordost health insurants in 2012. However, strong regional variations were observed, ranging from 2.7% in the south of Brandenburg up to 11.7% in the commuting-belt, surrounding Berlin (Fig 1). Clusters were detected mainly in west-Berlin, the commuting-belt, the northern parts of Brandenburg and the northwestern part of Mecklenburg-West-Pomerania. The spatially structured component explained 89.5% of the BYM model. The choice of prior and precision parameters had no detectable effect on the posterior estimates, indicating that the amount of data is large enough not to be influenced by prior assumptions about the distribution of the outcome.

### Demographic and socio–economic risk factors of COPD

We identified four variables, which explained 20.6% (adj. R^2^: 0.206) of the spatial variation of COPD prevalence in a global Ordinary-Least-Squares model: (i) the proportion of insurants aged 65 and older, (ii) proportion of insurants with migration background, (iii) area deprivation and (iv) household size. Multicollinearity was very low among the explanatory variables, as indicated by the global VIF (Table 3). However, the global model performed very poorly in terms of the explained variance and clustering of the residuals.

**Table 3:** Results of the global OLS model. Significance levels: * <0.05, ** <0.01. ***<0.001.

Variable	Coefficient	VIF
**intercept**	0.0627*	
**Insurants aged 65 and older (%)**	0.020***	2.2456
**Insurants with migration background (%)**	0.033*	2.0878
**Area deprivation**	0.003*	1.7968
**Household size**	0.408**	1.5766
**Adjusted R**^**2**^	0.206	
**AICc**	-0.6	
**Global Moran`s I of residuals**	I = 0.424 (p<0.001)	

### Spatially varying risk factors of COPD

**Fig 2:**
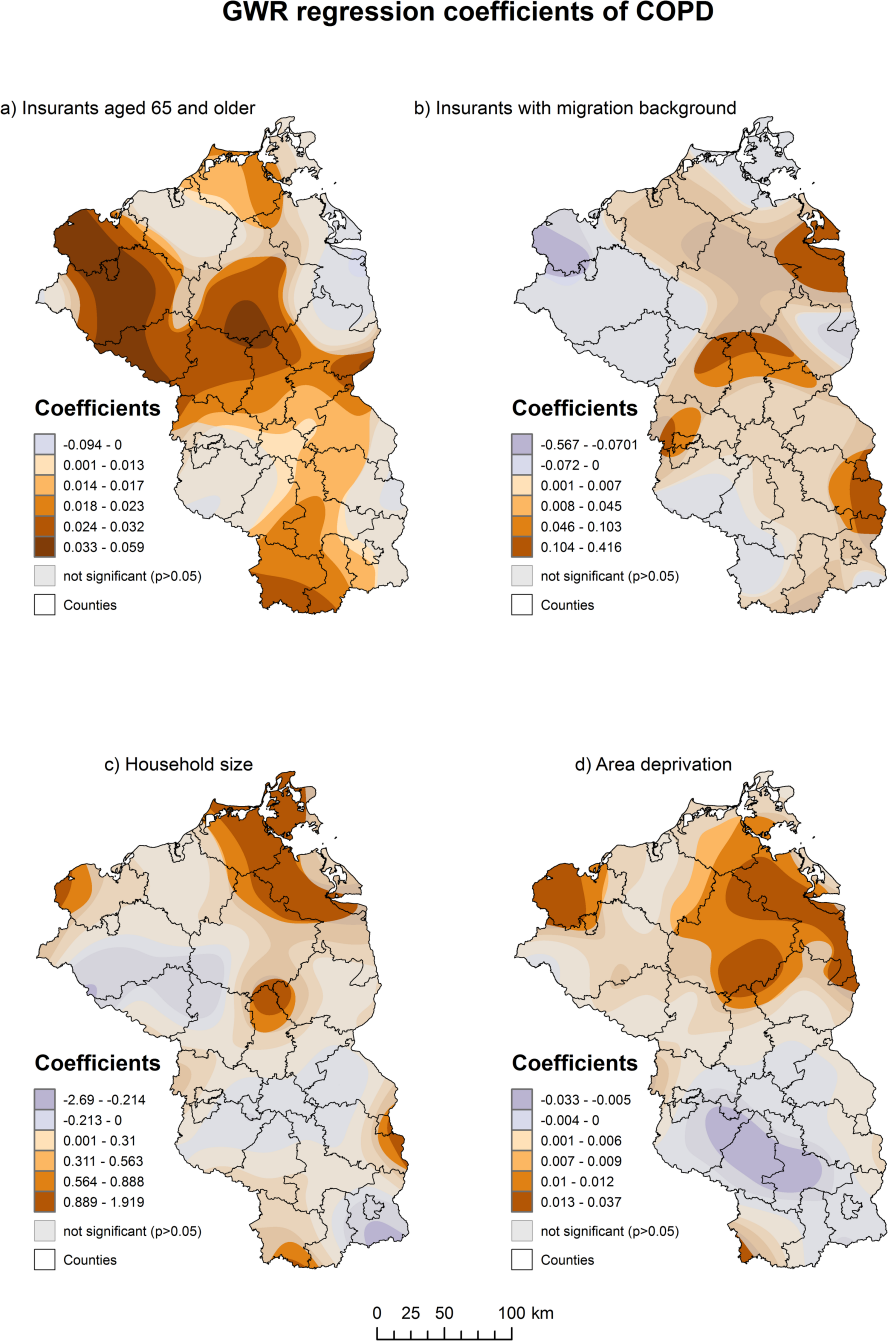
GWR regression coefficients of COPD.

Of the 20 evaluated combinations of kernel form, bandwidth type and optimization method, the GWR model with a Gaussian kernel form and a fixed, CV-optimized bandwidth had the best model fit among the models fulfilling the requirements of the residuals not being spatially autocorrelated (Table 4). This model outperformed the global model by far (AICc = -111.5) and explained 55% of the spatial variation of COPD prevalence (adjusted R^2^ = 0.551). The associations between COPD prevalence and the identified risk factors display strong regional variations and none of the predictors was significant in the entire study region (Fig 2).

**Table 4:** Evaluated combinations of kernel function, bandwidth type and optimization method for COPD. Significance levels: * <0.05, ** <0.01. ***<0.001.

Kernel type	Bandwidth	Optimization	AICc	Adjusted R^2^	Moran`s I
Gaussian	adaptive	AICc	-97.9	0.459	0.156***
Bisquare	adaptive	AICc	-89.3	0.464	0.142***
Exponential	adaptive	AICc	-99.8	0.454	0.185***
Tricube	adaptive	AICc	-87.7	0.460	0.147***
Boxcar	adaptive	AICc	-78.9	0.420	0.186***
Gaussian	fixed	AICc	-113.2	0.543	0.079*
Bisquare	fixed	AICc	-110.1	0.529	0.082**
Exponential	fixed	AICc	-107.6	0.500	0.134***
Tricube	fixed	AICc	-109.1	0.528	0.079*
Boxcar	fixed	AICc	-94.4	0.551	0.049
Gaussian	adaptive	CV	-97.9	0.459	0.157***
Bisquare	adaptive	CV	-74.9	0.522	0.075*
Exponential	adaptive	CV	-99.8	0.454	0.185***
Tricube	adaptive	CV	-79.9	0.509	0.092**
Boxcar	adaptive	CV	-78.9	0.420	0.187***
Gaussian	fixed	CV	-111.5	0.551	0.056
Bisquare	fixed	CV	-107.6	0.506	0.113***
Exponential	fixed	CV	-82.9	0.563	0.061*
Tricube	fixed	CV	-89.6	0.579	0.007
Boxcar	fixed	CV	-75.2	0.400	0.238***

The strongest impact of the proportion of insurants aged 65 and older was observed in Mecklenburg West-Pomerania. One percent increase in insurants aged 65 and older will increase the prevalence of COPD in this area by 3.3–5.9%.

The association between COPD and the proportion insurants with migration background was only significant around several cities such as Rheinsberg, Brandenburg an der Havel, Frankfurt (Oder) and Ueckermünde. A 0.1% increase of insurants with migration background will increase the prevalence of COPD in these areas by 1–4%.

Household-size was only significant in a fraction of the study area. The strongest and significant impact could be observed in the northern part of Brandenburg, Frankfurt (Oder) and the northern parts of Mecklenburg-West-Pomerania. An increase of 0.1 persons per household will increase the prevalence of COPD by 0.8–1.9% in these areas.

Area deprivation was only significantly positive associated with COPD in several parts of Mecklenburg-West-Pomerania. An increase of one point on the deprivation score will increase the prevalence of COPD by 1.3–3.7%. A significantly negative association could be observed south of Berlin in the commuting belt.

## Discussion

This is the first study in Germany to analyze the spatial distribution of COPD at the smallest spatial scale possible. The sex- and age-adjusted prevalence varies considerably across northeastern Germany and clusters especially in Berlin, it`s surrounding municipalities and the northern parts of Brandenburg. The risk factors for COPD are proportions of insurants aged 65 and older, foreign insurants, household size and area deprivation.

The raw prevalence of 8.3% and the sex- and age-adjusted prevalence of 6.5% is in the range of current prevalence estimates for COPD in Germany, although it should be noted that a direct comparison is not suitable due to different study designs and smaller sample sizes [5, 9, 11, 13]. When compared to GIS-based studies in other countries, such as Canada [15], the US [40] and the UK [41], our prevalence estimates still remain in the range of these studies. This is to an extent surprising, given that the prevalence of chronic diseases is typically higher among members of the AOK [17–20]. We would have therefore expected a higher prevalence rate in our database.

The prevalence estimates varied strongly between the municipalities and urban districts and showed strong local clustering. The highest prevalence rates were observed in West-Berlin, smaller parts of Brandenburg, but were mostly below average in Mecklenburg-West-Pomerania. Strong regional differences and local clustering not only for COPD [15, 23], but also for other respiratory diseases such as asthma [14] is typical and our results provide further evidence for local clustering of COPD. The large difference between Berlin and surrounding rural areas may be explained in part by the fact that persons living in urban areas are more likely to smoke [42, 43] and a higher exposure of inhabitants in Berlin to traffic-related air pollution. Generally, the prevalence of COPD follows–to an extent–the proportion of smokers in Germany [43].

In this study, we identified proportions of insurants aged 65 and older, insurants with migration background, household size and area deprivation as significant predictors for COPD.

The association of COPD to insurants aged 65 and older is not surprising, given the strong association between COPD and advanced age groups [4, 44]. However, our results demonstrate that the association between COPD and proportion of insurants aged 65 and older is not everywhere significant and varies considerably across northeastern Germany. We could observe a stronger association especially in more socially disadvantaged areas. The finding that demographic variables display a stronger association to chronic diseases in more disadvantaged areas has been noted in several studies applying GWR [24, 45]. Several studies focusing on the effect of area deprivation on health have reported that persons aging in socially disadvantaged neighborhoods are at higher risk for chronic diseases, irrespective of their socio-economic characteristics at the individual level [28, 46–48]. The partial similarity between the index of regional deprivation and the coefficients for the proportion of insurants aged 65 and older reflects the findings of these studies, although not as pronounced as for hypertension [24].

The proportion of insurants with migration background was the second risk factor specific to the composition of members of the AOK Nordost. Several studies in the US found that migrants are a risk-group for COPD [49, 50]. A study in the Netherlands concluded however, that the burden of COPD among non-western immigrants is lower than among the Dutch population. Although the overall prevalence of COPD in our database is similarly lower among insurants with migration background than among German insurants, the areas highlighted by the GWR analysis for the coefficients of insurants with migration background are the areas where the proportion of unemployed migrants is specifically high. Given the association between social status and smoking [51], it is therefore not surprising that insurants with migration background were only significantly associated in areas where the proportion of unemployed migrants is high, but not in the areas where the general proportion of migrants is high such as Berlin and surrounding areas. This finding underlines the value of a local regression approach as a global association to the proportion of insurants with migration background would be dismissed as implausible due to the lower overall prevalence rate of COPD in migrants.

Household size was an important predictor relating to household characteristics. Although a positive association to household-size may be considered as counter-intuitive at first, given the higher proportion of smokers in one-person-households [51], it is important to see household size—at least in part of the study area—in relation to migration background. In Germany, the average household size among migrants is higher than among Germans [52]. Based on the results of GWR, there seems to be an association between the prevalence of COPD and unemployed insurants with migration background living in larger households. This is reflected by the similar distribution of the coefficients for insurants with migration background and household size, which overlap especially in the northern municipalities of Brandenburg, Frankfurt (Oder) and Ueckermünde. It is however important to note, that household size is not always correlated to the proportion of insurants with migration background. In Rügen, household size was positively associated with COPD, although the proportion of insurants with migration background was not significant in this area. It has been pointed out that persons residing in a living community or shared appartment and unmarried persons in a steady relationship comprise an important risk group for smoking in Germany [53]. Possibly, the positive relationship to household-size in Rügen reflects this finding although further research on an individual level should confirm this association.

Area deprivation was mainly only in Mecklenburg-West-Pomerania significantly positive associated with COPD. In the southern part of the commuting belt, this association was significantly negative. Previous studies have reported an adverse relationship between COPD and socio-economic status [50, 54]. Our study adds a new level of detail to previous studies as it highlights not only that lower socio-economic status has only a significant impact in parts of the study area but also that in the commuting belt, a higher SES may be also associated with COPD. This finding is in line with previous studies applying GWR for the analysis of the association between chronic diseases and area deprivation [24, 45] and reflects the findings for the commuting belt around Berlin of a previous study [24].

### Implications for planning of healthcare and prevention

There are concerns that the current target ratio of 1671 inhabitants per GP at the scale of central areas (Mittelbereiche) is too simplified and does not necessarily reflect the actual demand for healthcare [24, 55]. Although the association between area deprivation and health can already be considered as established in the international literature [45, 48, 56–60], similar findings in Germany have been published only in more recent years [24, 28, 29, 61, 62]. Area deprivation had in this study only a significant and therefore direct effect in a small part of the study area. It`s indirect effect can be however seen by the partially similar pattern between the GIMD and the coefficients for persons aged 65 and older. This similarity reflects previous results that persons aging in socially disadvantaged regions have a higher chance of developing a chronic disease, even when they are not directly affected by deprivation on an individual level such as being unemployed or having a low income [24, 28, 46, 48]. Although the association between area deprivation and health outcomes can slowly be considered as established also in the German context, the current guidelines of the federal association of statutory health insurance physicians still rely only on the above mentioned target ratio and do not acknowledge a higher demand for healthcare in socially disadvantaged regions by default. However, these guidelines would allow deviations from this ratio for areas with statistically significant increased prevalence rates or specific socio-economic area characteristics [63]. We have clearly detected such areas in Berlin, it`s surrounding commuting belt and several parts of Brandenburg and Mecklenburg-West-Pomerania. Additionally, the association to area deprivation, which has been also demonstrated for other diseases in our study area [18, 24] clearly demands the inclusion of area deprivation into planning of healthcare.

To possibly prevent or alleviate a further increase of COPD, preventive actions will be necessary. The areas highlighted as local clusters could serve as a first basis to prioritize future preventive actions. The results of the GWR analysis clearly point out that persons aging in socially disadvantaged areas are possibly at higher risk of developing COPD, irrespective of their individual socio-economic characteristics. Additionally, migrants in multi-person-households residing in socially disadvantaged areas in the northern municipalities of Brandenburg, Frankfurt (Oder) and Ueckermünde are possibly at higher risk of smoking and therefore developing COPD. These results could be used to implement cost-effective prevention strategies aimed at the location-specific risk groups identified in this study. Similar approaches have been applied to target location-specific risk groups for various diseases such as cardiovascular disease [45], coronary heart disease [64], type 2 Diabetes Mellitus [16, 18, 20, 65] and Hepatitis C [66]. It is however important to stress that our results account only for insurants of the AOK Nordost and are not representative for the total population of northeastern Germany. It would be desirable to evaluate, in how far the results would differ if our analysis would be repeated for all statutory health insurants. However, such a comparison is unlikely to happen anytime soon as data for all statutory health insurants is generally only available at the scale of counties in Germany [67]. This scale is however, very coarse and the level of in-area variation is too high for meaningful prevention strategies and planning of healthcare at the very local level [61].

### Strengths and limitations of this study

#### Strengths

First, we used a large database consisting of 1.8 million individual insurants. Our results are therefore representative for a quarter of northeastern Germany`s population.

Second, we geocoded the health insurance claims for the disease mapping approach and cluster analysis to the smallest administrative units available and for the regression analysis to the second-smallest administrative unit in Germany. Most spatial epidemiological research in Germany is still conducted at the county-level [55, 61, 67, 68], although the large variation within counties increases the likelihood of ecological fallacy [61]. Our results therefore add a new level of detail to current spatial epidemiological research in Germany.

Third, our results clearly demonstrate that a local spatial regression approach is by far more useful than a global regression approach. The results of GWR clearly displayed, which population group in specific locations is at risk for COPD.

#### Limitations

First, although this study relied on a very large database of northeastern Germany`s biggest health insurance provider, the results are only representative for members of the AOK Nordost, but not for the total population. Given the lack of spatial epidemiological studies related to COPD, it currently remains unknown in how far our results would differ from other health insurance providers.

Second, the proportion of AOK Nordost insurants is higher in more deprived areas and lower in less deprived areas. Logically, our prevalence estimates are biased towards socially disadvantaged areas. As a result, the association of COPD to area deprivation is possibly alleviated in our population sample. In how far our results deviate from the prevalence of COPD in the general population could not be evaluated, given the lack of spatial epidemiological studies on COPD in Germany.

Third, only the diagnosis of COPD was available within the database. However, the actual severity of COPD as indicated by the Global Initiative for Chronic Obstructive Lung Disease (GOLD) classification [69] was not available within the database. In how far the treating physician was able to correctly diagnose COPD could not be evaluated. Possibly, there is an over-diagnosis among elderly persons if the GOLD classification was applied [70]. In contradiction, several studies found that COPD is often underdiagnosed, with sometimes only 20–30% of persons being correctly identified as having COPD [71]. A study in England estimated that only 52% of the expected COPD cases are diagnosed [41]. In how far the prevalence within the health insurance claims really reflects the actual prevalence is therefore unknown.

Fourth, it is clear that smoking still remains the main risk factor for COPD [6]. However, this information is unavailable in the insurance database. Our analysis therefore misses one of the most important determinants of COPD.

Fifth, a wide range of studies using GIS for the analysis of COPD focus on the effect of traffic-related air pollution on the occurrence of COPD [72–74]. During the initial data analysis, we aimed to include a measure for traffic-related air pollution in the model as well. However, the currently available data on fine particulate matter from Germany`s federal environmental office are based only on 375 measurement stations for Germany. Since fine particulate matter concentrations fall already at a distance of 400m from the source to normal background levels [75], the spatial resolution of the data from the federal environment office was considered too coarse. We also retrieved data from the federal highway research institute to estimate traffic load for northeastern Germany similar to previous studies [73]. However, traffic load was only available for selected streets, but not all and did not include several main roads and highways of possible interest. We therefore chose not to use this data source. Our last approach of analyzing possible associations between COPD and traffic-related air pollution was to use the proportion of insurants living <100m [73] to the nearest highway or main road based on data from OpenStreetMap. Although this variable was significantly associated with COPD, the results of the GWR analysis revealed mainly associations in rather remote rural areas, where such associations seem implausible. In contradiction, in Germany`s largest urban area Berlin, this association was not significant. We therefore had to decide that an investigation about the effect of traffic-related air pollution on COPD is currently not feasible with the available data.

Sixth, the administrative structure of Germany complicates spatial epidemiological research. The smallest administrative units, for which demographic and socio-economic data are available, are municipalities. However, large cities such as Berlin with 3.5 million inhabitants count as only one single municipality. As a result, the likelihood of ecological fallacy is higher in and around large cities as compared to rural areas. Also, the municipalities vary greatly in size and inhabitants between Brandenburg and Mecklenburg-Vorpommern. We would have favored to conduct the spatial regression analysis also at the smallest spatial scale. However, the municipalities in Mecklenburg-West-Pomerania are by far smaller than in Brandenburg. This creates a problem for spatial regression analyses as the residuals remain always clustered in the border region between Brandenburg and Mecklenburg-West-Pomerania, irrespective of kernel distribution and bandwidth size of the GWR analysis. For the future, it would be highly desirable to have administrative units, which are comparable in their number of inhabitants to be able to analyze intra-urban differences as well.

Seventh, although the methodology behind GWR has improved within the last years with various diagnostic criteria available [38], Wheeler et al. see the use of GWR only as exploratory but not as inferential. This is partly due to the subjectivity of the choice of bandwidth and issues arising from estimating the local parameters based on several local regression equations rather than one single regression equation [76]. We tried to alleviate the issue of subjective choice of bandwidth by evaluating all possible combinations of kernel distribution, kernel size and optimization method based on their AICc value, adjusted R^2^ and clustering of the residuals. However, we do have to agree that the local coefficients should be rather treated as estimates and not inferential values. In comparison to a global model however, we see one single coefficient per explanatory variable as highly unrealistic and implausible. This is reflected by the poor performance of the global model as well. Logically, the use of GWR highly improved the interpretational utility of spatial regression modelling, even when the coefficients should only be treated as estimations.

## Conclusions

This is currently the first and most detailed spatial epidemiological study of COPD in Germany. Our results clearly display that the prevalence varies at the very local level. The association to area deprivation not only for COPD but also for other common chronic diseases requires the incorporation of area deprivation into planning of primary healthcare in Germany. The spatially varying associations between insurants aged 65 and older, insurants with migration background, household size and area deprivation provide a useful starting point for future prevention strategies by pointing out who is where at risk for COPD.

## Abbreviations

AICc: Akaike`s corrected Information Criterion
AOK: Allgemeine Ortskrankenkasse
BBSR: Federal Agency of Building and Urban Development
BYM: Besag–York–Mollié model
COPD: Chronic obstructive pulmonary disease
CV: Cross validation
GIMD: German Index of Multiple Deprivation
GOLD: Global Initiative for Chronic Obstructive Lung Disease
GP: General practitioner
GWR: Geographically weighted regression
ICD: International classification of disease
INLA: Integrated nested Laplace approximation
Km: Kilometer
OLS: Ordinary least squares
VIF: Variance inflation factor
WHO: World Health Organization
